# Skiagraphic Atlas of Fractures and Dislocations, with Notes on Treatment, for the Use of Students

**Published:** 1900-03

**Authors:** 


**Skiagraphic Atlas of Fractures and Dislocations, with Notes on Treatment, for the Use of Students.** By Donald J. Mackintosh, M.B. London: H. K. Lewis. 1899.

When we find included in this book skiagrams of a bullet in the chest, a needle in the hand, &c., we are inclined to think that the title "Skiagraphic Atlas of Fractures and Dislocations" is somewhat of a misnomer. As a record of the everyday X-ray work of a large general hospital it is excellent, but hardly to be dignified by the title of an atlas.

Like all other series of skiagrams, some prints are better than others.

We think it is a mistake to introduce the question of treatment into a book of this sort, for a description of treatment must necessarily be perfunctory. Take the treatment for Colles's fracture for instance. After very properly insisting on reduction of the fracture, the directions given to put up the injured part on "an anterior junk splint" would not convey much information to a student who had not been educated at the Western Infirmary, Glasgow. If the book had been kept exclusively to X-ray work, and some more details given about the means used in producing the skiagrams (such as length of spark, whether hard or soft tubes were used, &c.), we think its value would have been materially increased.

The "get up" of the book is very good indeed, and the reproduction of the plates leaves nothing to be desired.

